# Spatial Resolution of Suprachoroidal–Transretinal Stimulation Estimated by Recording Single-Unit Activity From the Cat Lateral Geniculate Nucleus

**DOI:** 10.3389/fnins.2021.717429

**Published:** 2021-10-13

**Authors:** Tomomitsu Miyoshi, Takeshi Morimoto, Hajime Sawai, Takashi Fujikado

**Affiliations:** ^1^Department of Integrative Physiology, Graduate School of Medicine, Osaka University, Suita, Japan; ^2^Department of Applied Visual Science, Graduate School of Medicine, Osaka University, Suita, Japan; ^3^Graduate School of Nursing, Osaka Prefecture University, Habikino, Japan; ^4^Graduate School of Frontier Biosciences, Osaka University, Suita, Japan

**Keywords:** retinal prosthesis, suprachoroidal–transretinal stimulation, lateral geniculate nucleus, single-unit recording, retinitis pigmentosa

## Abstract

Retinal prostheses are devices used to restore visual sensation in patients suffering from photoreceptor degeneration, such as retinitis pigmentosa. Suprachoroidal–transretinal stimulation (STS) is a prosthesis with retinal electrodes located in the sclera. STS has the advantage that it is safer than epiretinal or subretinal prostheses, as the implant is not directly attached to the retinal tissue. We have previously reported feasibility of STS with animal experiments and clinical trials. However, functional evaluation with neurophysiological experiments is still largely missing. To estimate the spatial resolution of STS, single-unit activities in response to STS were recorded from relay cells in the dorsal lateral geniculate nucleus of cats, and the response probability of the units was analyzed in relation to the distance between the stimulus location and the receptive field of each recorded unit. A platinum electrode was attached to the sclera after lamellar resection, and the return electrode was placed in the vitreous. The stimulating current, which ranged from 50 to 500 μA, was applied between these electrodes, and the probability of spike responses occurring just after retinal stimulation was measured. The distance at half-maximum of response was determined from the collected response probabilities as a function of stimulus intensity for all units characterized by their distances from the receptive field center to the stimulation point. As the stimulation became weaker, this distance decreased to 1.8° at 150 and 100 μA. As another estimation, the radius of 25% response probability was 1.4° at 100 μA. The diameter of the stimulated cat retinal area, 3.6° or 2.8°, corresponds to human visual acuity of 0.005 or 0.007, or finger counting. Considering the lower hazard to the retina of STS and its potentially large visual field coverage, STS is an attractive method for retinal prosthetic device development.

## Introduction

Retinitis pigmentosa (RP) is the leading cause of blindness and is characterized by the degeneration of photoreceptors (Marmor et al., 1983; Pagon, 1988). However, at present, there is no effective treatment. As a biomedical engineering approach to restore vision in these patients, retinal prostheses have been intensively studied (for review, Ayton et al., 2020). In retinal prosthesis, the residual retinal neurons of RP patients are electrically stimulated by an implanted electrode array to detect light sensation, called “phosphenes.”

Two types of retinal prostheses have been developed thus far: epiretinal stimulation (Humayun et al., 1994; Majji et al., 1999; Nadig, 1999; Walter and Heimann, 2000) and subretinal stimulation (Chow and Chow, 1997; Zrenner et al., 1999; Schwahn et al., 2001; Chow et al., 2002), named according to the location of the electrode array implantation. Both approaches have a common disadvantage, in that, the stimulating electrode array is invasive for the neural retina because it is directly attached to the retina. Although improvements in surgical and electronic technology may solve some of the problems associated with these types of retinal prostheses, the potential risk of damage to the eye after intraocularly inserting an electrode is still debatable.

To minimize the invasion of the retina by the stimulating electrode, our group previously developed an original stimulating method named suprachoroidal–transretinal stimulation (STS), in which the electrode array is placed in the scleral pocket or the suprachoroidal space (Kanda et al., 2004; Sakaguchi et al., 2004; Nakauchi et al., 2005). This design minimizes the retinal insult because the stimulating electrode array does not attach directly to the retina. In exchange for this advantage, the electrode is located farther from the retinal neurons than the other two types, and this array location might reduce the resolution of the prosthesis. For human patients, both our STS and a suprachoroidal implant by Bionic Vision Australia improved the patients’ vision-related behavior (Fujikado et al., 2011, 2016; Ayton et al., 2014; Barnes et al., 2016), but it is still difficult to assess the resolution limit of suprachoroidal stimulation because of restrictions in human studies. Thus, it is important to investigate the spatial resolution limit that can be achieved through this retinal prosthesis for activation of the retino-geniculo-cortical pathway of an animal model such as the cat, which has a well-developed visual system.

Single-unit response elicited by STS was recorded from the relay cells of the dorsal lateral geniculate nucleus (dLGN). The spatial extent of the activated area was estimated using the spike response of each unit and the distance between its receptive field (RF) and the stimulating electrode. The spatial resolution of suprachoroidal stimulation has previously been reported from the responses of the cat visual cortex (for example, Shivdasani et al., 2012; John et al., 2013; Wong et al., 2016). Because the cortical visual processing modifies the input signal from the dLGN, the cortical responses are more characteristic of cognition. In contrast, the single-unit recording from dLGN reveals the responses closer to the activity of retinal ganglion cell (RGC) stimulated by STS. Here we investigated the threshold and the spatial extent of the activated retinal area.

## Materials and Methods

### Animal and Surgical Preparation

Twelve adult cats (either sex, weight 3–4 kg), bred at the Graduate School of Medicine, Osaka University, were used. All animal procedures were performed in accordance with the Guide for the Care and Use of Laboratory Animals by the Institute for Laboratory Animal Research, and the guidelines of the Graduate School of Medicine, Osaka University. Every effort was made to minimize animal discomfort and reduce the number of animals used.

After intramuscular injection of ketamine hydrochloride (25 mg/kg) and intraperitoneal injection of atropine sulfate (0.1 mg/kg), the cats were paralyzed and anesthetized by intravenous infusion of Ringer’s solution (0.9 mL/kg/h) containing pentobarbital sodium (1 mg/kg/h), pancuronium bromide (0.2 mg/kg/h), and glucose (0.1 g/kg/h). The animal was artificially ventilated *via* a tracheal tube with an N_2_O/O_2_ gas mixture (1:1). During the experiment, end-tidal CO_2_ concentration, intratracheal pressure, and electrocardiogram were continuously monitored. The body temperature was maintained using a heat pad at 38°C. The pupils were dilated with mydriatics, a mixture of tropicamide and phenylephrine hydrochloride (Mydrin-P, Santen Pharmaceutical Co., Ltd., Japan) and 1% atropine sulfate (Nitten Pharmaceutical Co., Ltd., Japan).

Bipolar stainless-steel electrodes were placed bilaterally beside the optic chiasm (OX) for stimulation. The position of the electrode tip was determined based on the flash-evoked response of the electrode.

For implantation of the STS electrodes, a skin incision was made horizontally approximately 20 mm from the lateral angle of the left eye. The temporal orbital bone was partially removed, and the lateral rectus muscle was dissected to expose the temporal surface of the eye. The upper temporal scleral area was located approximately 15 mm posterior to the corneal limbs, and just above the long ciliary artery was exposed. Subsequently, scleral lamellar resection (size: approximately 4 × 4 mm) was performed up to half depth using a razor blade or a crescent knife, and the STS electrode was attached by the manipulator (see section “Suprachoroidal–Transretinal Stimulation”). The conjunctiva around the corneal limb was sutured to an eye ring, which was attached to the head holder of the stereotaxic instrument, to prevent eye movement. A return electrode made of a urethane-coated platinum wire (200 μm in diameter) with the tip exposed by approximately 2 mm was inserted into the vitreous through the pars plana.

### Electrophysiological Recording

A glass-coated tungsten microelectrode (1–3 Mohm) was inserted stereotaxically into the A1 layer of the left dLGN to record the single-unit activities of relay cells. The electrical signal was amplified 2,000–10,000 times and filtered between 300 and 5 kHz using an AC amplifier (Model 1800 Microelectrode AC amplifier, A-M SYSTEMS, INC., United States) and a low-pass filter (LPF-202A, Warner Instruments, LLC, United States). The signal was monitored using an oscilloscope and audio monitor in real time. The amplified signal was acquired on a data acquisition interface (Power 1401, Cambridge Electronic Design, United Kingdom), with a sampling frequency of 50 kHz and analyzed offline using the software Spike2 (Cambridge Electronic Design, England) and MATLAB (The Mathworks, Inc., United States). To stimulate OX, monophasic pulses with a duration of 50 μs were delivered from a pulse generator (SEN-7203, Nihon Kohden, Japan) through an isolator (SS-202J; Nihon Kohden).

### Receptive Field Plotting

The RF center of each recorded unit was plotted on a tangent screen positioned 114 cm from the eye. On this screen, 2 cm was equal to 1° of visual angle. The eye was refracted using a contact lens to focus it on the screen. The RF was delineated by monitoring the electrical activity of the unit in response to the small test spot being turned on or off within the RF or in response to the movement of the spot in and out of the RF (Miyoshi et al., 1999).

### Identification of Cell Types

Each unit activity was classified into either Y-cells or X-cells of relay neurons, based on features such as light response, RF center size, OX latency, and response linearity of light input, as established in previous studies (Cleland et al., 1971; Hoffmann et al., 1972). The unit with (1) a phasic RF response to on or off stationary light stimulation, (2) a response to fast repetitive stimuli, and (3) a large RF center (0.6°–2.5°), was classified as Y-cell. On the other hand, when the unit had tonic response to stationary light stimulation, no response to fast repetitive stimuli, and a small RF center (0.1°–1.3°), it was classified as an X-cell. In some units, our classification was confirmed by a null test, which characterizes the input linearity with pattern-reversal stimulation by a digital projector (Enroth-Cugell and Robson, 1966; Takao et al., 2002). The latency in OX stimulation also helped to confirm the relay neuron and Y/X classification. When classification into either a Y-cell or an X-cell failed, the unit was excluded from further analysis. These unclassified units were rare and recorded mainly at the boundary region of the LGN A1 layer. In addition, we classified the included units into sub-categories: on-center/off-surround or off-center/on-surround.

### Suprachoroidal–Transretinal Stimulation

Two types of electrodes were used to stimulate the retina. One was a multichannel electrode array (Unique Medical, Japan), which had nine stimulation sites arranged in three-by-three grids with a center-to-center distance of 0.5 mm on the silicone base (size: 3 × 6 mm). The stimulation sites were made of platinum and had a diameter of 0.1 mm. The surfaces of the stimulation site protruded from the silicone base by 0.05 mm. The other was a single-channel electrode. The stimulation site was made with the same geometry and material as the multichannel electrode array. The silicone base of the single-channel electrode was round, 0.8 mm in diameter, and was set to a glass tube to be attached to the sclera with a micromanipulator. By pressing this single-channel electrode against the sclera strongly, the stimulation site was easily identified by a small bump in the funduscopy. Therefore, the single-channel electrode was used mainly to obtain the units with RF located very close to the stimulation point.

Between the scleral stimulating electrode and the vitreous return electrode, a single biphasic pulse of current (cathodic first without interphase interval, 0.5 ms) was applied at 1.0 s intervals *via* a linear isolator (BSI-950, Dagan Corporation, United States) connected to the data acquisition interface (Power 1401, Cambridge Electronic Design Limited, United Kingdom). The current intensity varied among 500, 300, 200, 150, 100, 70, and 50 μA. For each level, 40 stimulation trials were conducted.

### Measurement of Distance Between Receptive Field and Stimulation Point

Before the electrophysiological recording, the ocular fundus was back-projected onto the screen (Bishop et al., 1962; Fernald and Chase, 1971), and the retinal structures including the arteries, veins, and optic disc were traced on the tangent screen. Once the stimulating electrode was placed on the sclera, it was meticulously maintained at that position. After the recording, a small scar on the retina was created by applying a DC current (1 mA for 10 s) *via* the stimulating electrode to confirm the stimulation point on the retina. Subsequently, the eye was enucleated after the animals were deeply anesthetized with an overdose of sodium pentobarbital. The eyes were fixed with 10% formalin (Mildform 10N, Wako Pure Chemical Industries Ltd., Japan) for 30 min. The retina was isolated and flat-mounted on a glass slide, and the position of the scar, which indicated the stimulation point, was identified with other landmarks, such as vessel running and branching pattern.

The distances between the corresponding stimulation point and the central point of the RF center were measured on a tangent screen. The corresponding stimulation point on the screen was identified by overlapping the images of the flat-mounted retina and screen.

## Results

### Single-Unit Responses to Suprachoroidal–Transretinal Stimulation

**FIGURE 1:**
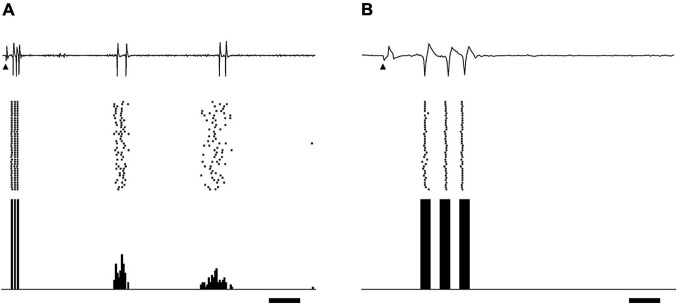
Example of relay cell response in dLGN evoked by biphasic pulses of STS. Extracellular recording waveforms (upper row), raster plots (middle row), and peri-stimulus time histograms (lower row) are shown. **(A)** STS of 300 μA evoked burst discharges on off-center Y-cells. The distance between the central point of the RF center and the stimulating electrode was 1.28°. Arrowheads indicate the stimulus onset. Scale bar, 20 ms. Panel **(B)** is an enlargement of panel A on the time axis to show the spikes in the first burst. The scale bar, 3 ms.

A total of 114 single-unit responses to STS were recorded from the dLGN of 12 cats (11 On-Y cells, 35 Off-Y cells, 28 On-X cells, and 40 Off-X cells). In most cases, the responses to STS consisted of several periodic bursts, up to 200 ms after the stimulus. Each burst consisted of fewer than six spikes. No spontaneous spike activity was observed during the silent period between bursts. A typical example of a single-unit response to an STS is shown in Figure 1. In this case, the response consisted of three burst discharges appearing at 4–10, 70–80, and 120–150 ms after STS.

These bursts were categorized into two types based on their latencies: the first burst and the late burst. The first burst was defined as a burst that appeared within 15 ms after the stimulus. The first burst consisted of spikes that were stable in their latencies and number (usually less than five spikes) under the same stimulus condition of the same unit (Figures 1A,B). This burst was rarely obtained when the stimulation point was located far from the RF, in particular for distance more than 10°, even if the current intensity was larger than 500 μA. On the other hand, the late burst was defined as a burst that periodically appeared more than 20 ms after the stimulus. The spikes in the late bursts varied in their numbers and latencies. We observed that many units far from the stimulus point did not show first bursts but only late bursts. The late response by epiretinal stimulation was reported to be derived from the presynaptic neurons of RGCs (Jensen et al., 2005a). The origin of the late bursts in the present study may also be retinal neurons before RGCs, but it is unclear from the current *in vivo* experiment. We do not know whether these late bursts were related to light perception in patients, and they did not seem to be associated with the location of the stimulus point. All these considerations led us to focus on first bursts rather than late bursts observed in the wide retinal area to evaluate the spatial properties of the STS in this study.

### Current Dependency of the Spike Discharges in the First Burst

**FIGURE 2:**
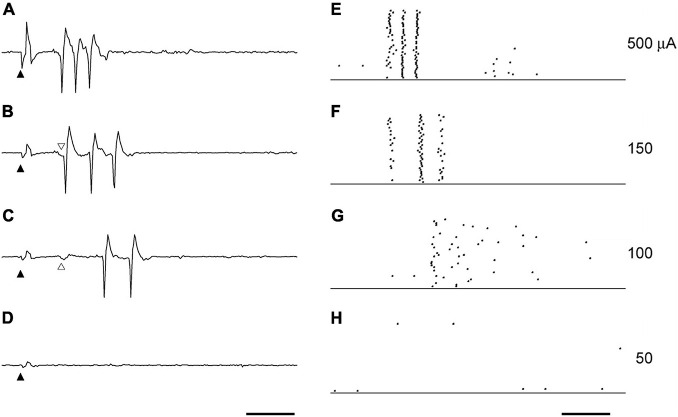
The effect of current intensity on the spikes triggers the first burst. The example waveforms **(A–D)** and the raster plots **(E–H)** by the STS of various intensities were obtained from the same unit to Figure 1. The current intensities were 500 μA **(A,E)**, 150 μA **(B,F)**, 100 μA **(C,G)**, and 50 μA **(D,H)**. Solid arrowheads indicate the timing of STS. Scale bars, 5 ms. Following the decrease in current intensity, the decrease of the spike discharges **(F,G)** an increase in the spike intervals **(E,F)** was observed. The latency of the first spike in **(A,B)** was not affected by the current intensity. The first spike usually followed the small deflection indicated by open arrowheads in **(B,C)**. This small deflection was still evoked with the same latency, even when the first spike disappeared with the STS of 100 μA **(C)**.

The spikes in the first burst were characterized by latency when the stimulation current was changed. The first spike in the first burst had a stable latency of 3–7 ms for most levels of stimulating current, whereas the spikes that followed the first spike were increasingly delayed as current stimulation was lowered. A representative example of the effect of the current intensity on the spikes in the first burst is shown in Figure 2. When the current intensity was 500 μA (Figures 2A,E), the three spikes regularly appeared with constant latencies of 4.47 ± 0.24 (mean ± SD of 40 trials), 5.89 ± 0.10, and 7.38 ± 0.11 ms. With 150 μA STS (Figures 2B,F), the three spike discharges appeared with latencies of 4.50 ± 0.17, 6.60 ± 0.05, and 8.27 ± 0.04 ms. The latencies of both the second and the third spike were prolonged, with decreases in the current intensities from 500 to 150 μA, whereas the latency of the first spike was not changed. At 100 μA, the first spike with a latency of approximately 4.5 ms disappeared, but a small deflection just before the first spike still existed, suggesting that the timing of synaptic input was preserved.

The first spike was observed in almost all the units when its RF was located near the stimulation point, and the current intensity was high. Y-cells tended to have a shorter latency in the first spike than X-cells. The late spikes usually consisted of two to five spikes, which had a latency of 5–15 ms. Following low intensity stimulation late spikes persisted with longer latency than for sufficiently strong stimulation, even when the first spike was missing.

### The Relationships Between Threshold Current Intensity and the Distance From Stimulating Electrode

**FIGURE 3:**
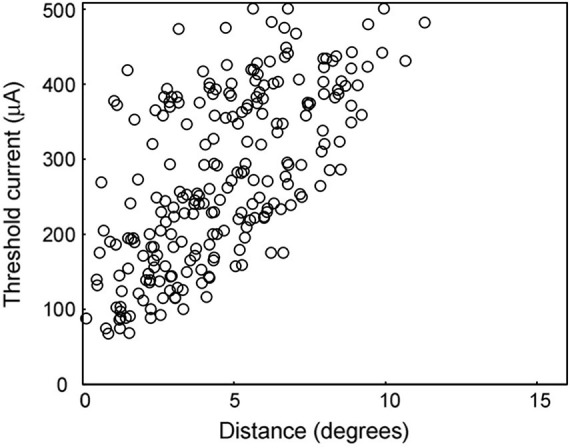
Threshold current intensity for 50% response probability. The distribution of this measure is shown for the distances between the RF center and the stimulating electrode. As the distance decreased, the threshold current tended to decrease.

First, we analyzed the relationship between the threshold of the current intensity for each recorded cell to evoke its first burst and the distance from the central point of its RF center to the stimulated retinal point (Figure 3), in order to evaluate the spatial extent of the neural responses to STS. The threshold current was defined as the electric current which generates a spike within 3–15 ms after stimulation (the period of the first burst) with a 50% probability out of 40 trials. The threshold was calculated using linear interpolation between the current intensity and the response probability of 40 trials.

The lowest edge delineated by this scattered plot shows the minimum threshold at that distance from the stimulation point. The area below this minimum threshold line indicates the range of currents that cannot excite the retina at that distance. The minimum threshold was 67 μA at a distance of 0°–2°, while it was 430 μA at a distance of 10°–12°. In the area over 12° from the stimulation point, there was no activated unit with a current less than or equal to 500 μA.

The threshold currents varied in each unit, even if they were located at the same distance from the stimulation point. In particular, a large variation in the threshold was observed for distances less than 5°. This indicates that every unit has a different threshold for electrical stimulation. However, the minimum thresholds tended to be lower at shorter distances, as mentioned above.

### The Spatial Extent of Neural Response to Suprachoroidal–Transretinal Stimulation Estimated by Response Probability Distribution

**FIGURE 4:**
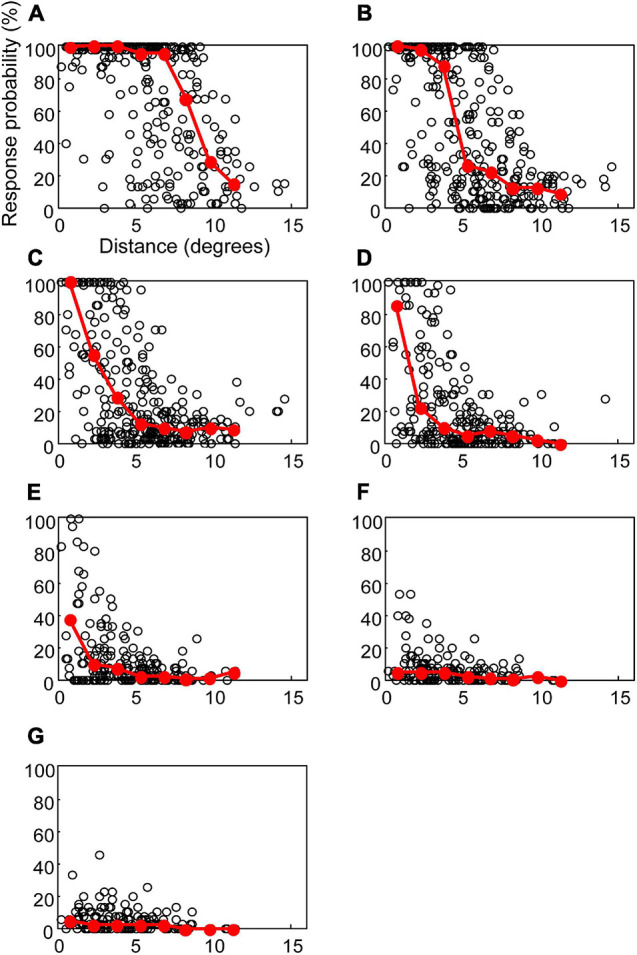
The response probabilities and their median values for the distances between the central point of RF and the stimulating electrode. Open circles indicate the response probability of each unit to the STS. Red circles and lines indicate the median of response probabilities for every 1.5°. The current intensities in **(A–G)** were 500, 300, 200, 150, 100, 70, and 50 μA, respectively.

The relationship between response probabilities and distances was analyzed to evaluate the spatial extent of the response to STS (Figure 4). The response probability was defined as the percentage of stimulation trials that resulted in a spike during the period of 3–15 ms after stimulation. The response probability increased when the RF center was located near the stimulation point. For example, after application of 200 μA (Figure 4C), response probability in excess of 80% was found between 0° and 5°. Thus, at that stimulus level the extent of the response to STS was limited to the vicinity of the stimulation point.

These results also showed significant variation in the response probabilities in the units located at the same distance from the stimulation point. In some units where the RF was located even less than 5° from the stimulation point, the response probability was lower than 5% at a current intensity of 500 μA. Although we categorized these data into the cell types (Y-cell or X-cell, and On-center cell or Off-center cell), we found no difference in distribution among the cell types (see detail in section “Discussion”).

The current intensity affects the distribution of response probabilities. At 500 μA (Figure 4A), the units with 80% response probability were found up to 10°. In the case of 150 and 100 μA (Figure 4D,E), the units with a response probability of over 80% were found within 5°. When the current intensity was lowered to 50 μA (Figure 4G), the units did not respond with a high-response probability, even if their RF centers were near the stimulation point. To evaluate the overall characteristics of the relationships between the response probability and the distance, the median of the response probability was calculated for every 1.5° interval and plotted in Figure 4. At 500 μA (Figure 4A), the medians lay on 100% from 0° to 7.5°. At 200 and 150 μA (Figures 4C,D), only the 0°–1.5° range showed a median value over 80%. These graphs of the medians indicate that the size of the high-response probability area depends on the current intensity.

**FIGURE 5:**
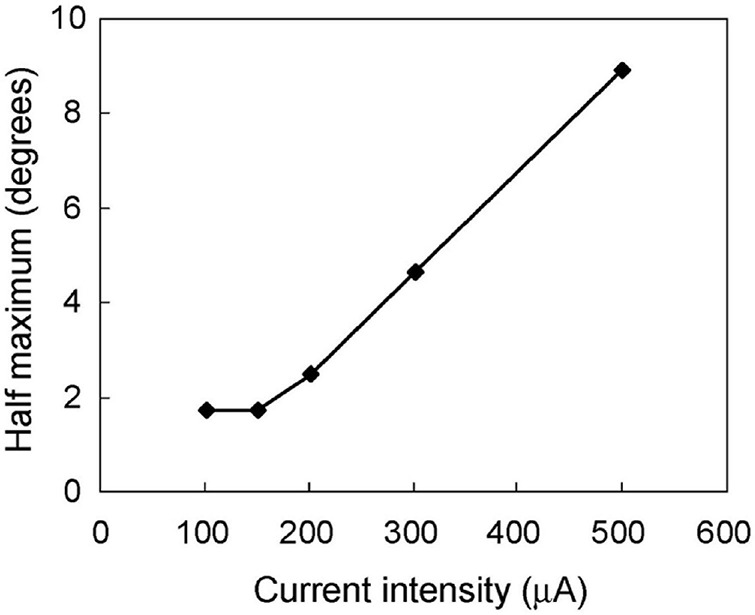
The relationship between the spatial extent of the neural response and the stimulus current intensity. The half width at half maximum was calculated from the median-distance graphs in Figure 4. The half width decreased following a decline in stimulus intensity from 500 to 150 μA. However, the half width was constant at 1.8° from 150 to 100 μA.

To investigate the relationship between the spatial extent of the activated area and the current intensity, the distance at the half maximum of the median plot was calculated for each simulation intensity. This distance was equivalent to half width at half maximum (HWHM) of the spatial extent of the retinal response by STS (Figure 5). The distances at half maximum at 70 and 50 μA were excluded because the responses were too weak. The value of HWHM was 8.9° at 500 μA, which decreased almost linearly with a decrease in the current intensity up to 150 μA, where it stabilized at 1.8°. The results indicated that a decrease in current intensity could localize the retinal responsive area.

**FIGURE 6:**
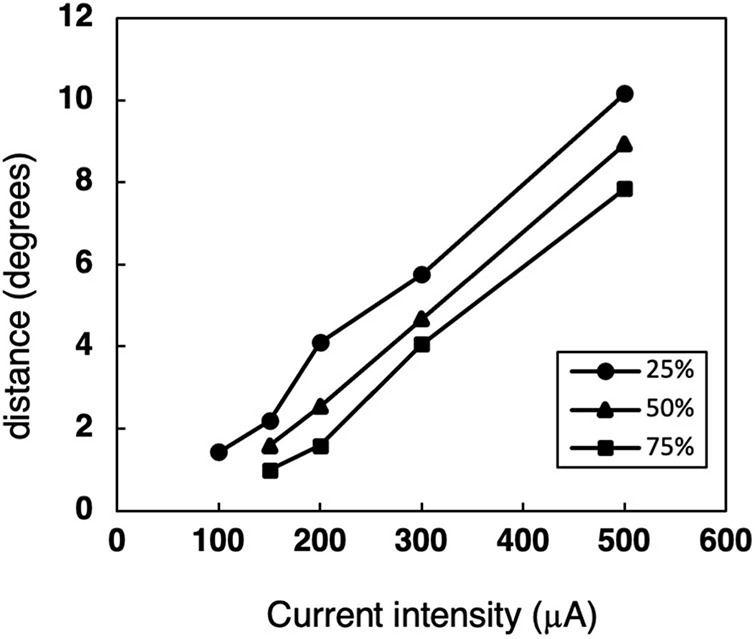
The relationship between the distance and current intensity with 75, 50, or 25% response probability thresholds. The data was calculated from the median-distance graphs in Figure 4.

To describe the relationships between the size of response area and the current intensity, the distances with 75, 50, or 25% response probability were also calculated from Figure 4 (Figure 6). The distance, radius of the activated area by STS, became smaller as the current decreased for the same response probability. With the smallest current, 100 μA, it was 1.4° for 25% probability.

## Discussion

### Burst Response by Suprachoroidal–Transretinal Stimulation in Dorsal Lateral Geniculate Nucleus Relay Cells

The responses to STS in the single-unit activity of dLGN were observed as several bursts, which repeated periodically up to approximately 200 ms after stimulus. A similar burst response to retinal electrical stimulation has been reported for RGC with epiretinal stimulation (Jensen et al., 2005b; Fried et al., 2006; Freeman and Fried, 2011), and transretinal stimulation (Crapper and Noell, 1963; Li et al., 2005). The activity of dLGN relay neurons followed the RGC activity by retinal stimulation in general, whereas the retino-geniculate connection dropped some spikes with weak retinal stimulation, as shown in the example of Figure 2C.

Although STS can theoretically stimulate all retinal neuron types, the first spike is thought to be caused by direct stimulation of the RGC. The antidromic response latency of cat RGCs to OX stimulation is about 1.5–2.5 ms in Y-cells and 3–5 ms in X-cells (Stone and Fukuda, 1974). OX latency of the cat dLGN relay cells is 0.9–1.8 ms in Y-cell and 1.5–3.1 ms in X-cell (Hoffmann et al., 1972). Thus, the latency of the dLGN relay neuron from the stimulated RGCs can be estimated approximately 2.4–4.3 ms in Y-cell and 4.5–8.1 ms in X-cell. This estimation corresponds well to the latency of the first spike in the first burst in response to STS. In an *in vitro* study of epiretinal stimulation, it was reported that the short latency spikes did not change their latency and still appeared after application of a synapse blocker; therefore, it was concluded that the spike represents the response to direct stimulation to the RGC (Jensen et al., 2005b; Fried et al., 2006). The feature of stable latency of the first spike by STS was similar to that of the short latency spike elicited by epiretinal stimulation. From these results, it was suggested that the first spike of the STS was elicited by direct stimulation of the RGCs, not *via* the retinal circuit before the RGCs. The latencies of the later spikes were more variable than those of the first spike. In an *in vitro* study of epiretinal stimulation, it was reported that the late spikes in the burst disappeared upon application of synaptic blockers (Fried et al., 2006). Further studies are necessary to discuss the appearance and mechanism of late spikes induced by STS.

### The Variation of Threshold Current

The threshold current intensity for 50% response probability had a wide variation, as shown in Figure 3. There are some possible reasons for this variability, and Figure 3 might be a mixture of thresholds caused by different features. To investigate the reasons, the threshold data of Figure 3 was classified according to the following four features: the cell type, the relative position of stimulating electrode, the electrode types, and the individual experimental animals (Supplementary Figures 1–4). As a result, all these features could not explain the wide variation of thresholds with a given retinal distance between the RF and the stimulating electrode.

The different types of neurons may have different excitability, which leads to the difference of the threshold. However, all four cell types, On-center/Off-center Y- and X-cells, still showed a wide distribution of thresholds (Supplementary Figure 1). The relative position of the stimulating electrode is important to the threshold because an electrode on an axon bundle can stimulate an axon directly. This may lower the threshold at a distant point from cell body and may deteriorate the topographic relation of retinal stimulation. We classified the threshold data according to the relative electrode position, nasal or temporal side from the central point of the RF (Supplementary Figure 2). Because the stimulating electrode is implanted on the temporal retina in this experiment, the stimulating electrode nasal to RF center had the possibility to stimulate the axon directly. In the scatter plot, the apparent drop of the threshold of the nasal electrode position was not found, and axonal stimulation cannot explain the variation. However, we do not think that STS can avoid axonal stimulation. Because the position of the stimulating array/electrode was fixed throughout the recording session, it may not be easy to sample the threshold of the stimulation on passing axons. This may be the reason why the difference did not appear, and we do not deny the possibility that STS activates the passing axons. Both the type of electrode (single electrode or electrode array) and the experimental session also could not explain the variation of the threshold (Supplementary Figures 3, 4).

To check the statistical contribution of these factors, we conducted a multiple regression analysis for the threshold. The five factors, Y-cell/X-cell, On-center/Off-center cell, the relative position of the stimulating electrode, the type of stimulating electrode, and the distance between RF and stimulating electrode, were used as explanatory variables. Among these five factors, the distance and the cell type (On-center/Off-center) are significantly effective to the objective valuable, whereas no significant effect was detected in the other three factors, namely the cell-type (Y-cell/X-cell), RF position (nasal/temporal to the RF center), and the electrode type (single/electrode array), as shown in Supplementary Table 1. Then, a regression analysis with a step-down procedure was performed with these two factors as explanatory variables, a significant prediction formula was performed, the coefficient of determination and coefficient of determination with adjusted degrees of freedom did not noticeably improve (Supplementary Table 2). The absolute value of the standardized regression coefficient (beta) was 11.756 for the retinal distance, being much larger than that for the cell type (On-center or Off-center cells). The latter value (beta) was −2.346, suggesting that Off-center cells were apt to respond with lower stimulus current than On-center cells.

In conclusion, these factors mentioned above cannot explain the wide variation of the threshold current. We may have to assume that there is an uncontrolled condition in relation to the neuronal excitability or the current spread; unfortunately we cannot identify it from the current data.

Threshold current of cortical multiunit activity for suprachoroidal monopolar electrode was previously reported as 173 ± 17 nC (346 ± 34 μA for our experiment) (Spencer et al., 2016). The corresponding thresholds in our experiment are those with the stimulating electrode near the RF, varying from 67 μA to approximately 400 μA, and almost all thresholds are lower than those recorded in the primary visual cortex. This difference in threshold suggests that the cortical threshold is modified and further processed from that of the RGCs through the visual information processing toward cognition.

### Spatial Extent of the Responsive Area Evoked by Suprachoroidal–Transretinal Stimulation

The present study showed that the HWHM of the minimum responsive area was 1.8° in the cat visual field by low-strength STS, which is equivalent to 0.40 mm of the retina (calculated from Vakkur et al., 1963). This HWHM of 0.40 mm conforms to 1.4° of the visual field in humans (3.5°/mm calculated from Oyster, 1999), assuming that the size of the responsive area was the same between the human retina and the cat retina. If two-point discrimination is achieved in the separation of HWHM in the retina, visual acuity can be 0.01. If the separation is required to be full width at half maximum (FWHM), the visual acuity can be half of 0.01, that is, finger counting.

From the area size under the same response probability (Figure 6), the distance with minimal current of 100 μA under 25% response probability was 1.4°. This corresponded to 1.1° in human, then the diameter of 25% response area was 2.2°. This means the visual acuity of 0.007, which is similar range to those from HWHM of the responsive area.

For epiretinal or subretinal stimulation, several groups reported distribution of responses in an *in vivo* study on the cat visual cortex. Local field potential (LFP) after retinal stimulation indicated that the FWHM of the responsive area was 1.49° to epiretinal stimulation (Wilms et al., 2003) and 1° to subretinal stimulation (Sachs et al., 2005). In addition, optical recording of the cat visual cortex (Eckhorn et al., 2006) showed that the average FWHM of the distribution was 1.28–1.29 mm on the visual cortex with epiretinal or subretinal stimulation, which corresponds to approximately 2.5°. The FWHM responsive area of 3.6° or the 25% response diameter of 2.8° from dLGN in the present study is a little larger than the aforementioned epiretinal and subretinal studies. With the suprachoroidal electrode, the size of the local field response on the cat visual cortex was reported to be 1.6°–2.7°, similar to the present results (Wong et al., 2016). This consistency with previous animal studies indicated that STS is comparable to other retinal prostheses in terms of resolution.

### Feasibility of Suprachoroidal–Transretinal Stimulation for Retinal Prosthesis

In STS, the stimulating electrode array is chronically implanted into the scleral pocket (Nakauchi et al., 2005; Fujikado et al., 2011, 2016), or between the sclera and choroid (Sakaguchi et al., 2004). This array position prevents physical damage to the retinal tissue in the long-term, as the scleral tissue supports the electrode array tightly. This stability of the electrode array can also maintain the distance from the electrode to the retina in the STS constant through long-term implantation. An *in vitro* study showed that the threshold was strongly affected by the distance from the electrode to the retina (Jensen et al., 2005b), indicating that a large variation in the distances can influence the quality of vision of the retinal prosthesis. Therefore, STS can maintain the quality of artificial vision, although the size of each phosphene might be larger than that of epi- or subretinal stimulation. In addition, a wider visual field can be achieved using a large array or multi-array (Lohmann et al., 2016).

In human preclinical studies of STS, patients reported that phosphene was stable and reproducible, and that the size of phosphene varied from the size of a pea to that of a quarter coin at arm’s length (Fujikado et al., 2011). This size roughly corresponds to 1°–2.5°of visual angle. Our present result, 2.2° or 2.8° in human terms, was in good agreement with the patients’ descriptions. Thus, our method to evaluate the extent of the retinal prosthetic response based on retinogeniculate projections is useful as a model for physiological studies of retinal prostheses. This would also be applied to studies that improve the spatial properties of prosthetic systems by stimulating parameters such as electrical waveforms and electrode combinations (for example, John et al., 2013; Dumm et al., 2014; Weitz et al., 2015; Spencer et al., 2016, 2017; Nakano et al., 2018).

## Data Availability Statement

The raw data supporting the conclusions of this article will be made available by the authors, without undue reservation.

## Ethics Statement

The animal study was reviewed and approved by the Animal Research Committee of the Graduate School of Medicine, Osaka University.

## Author Contributions

HS and TF designed the research and reviewed the manuscript. TMi and TMo performed the experiments. TMi wrote the manuscript. All authors discussed and approved the submitted version.

## Conflict of Interest

TMo and TF received financial support from the development partner, Nidek Co., Ltd. The funder was not involved in the study design, collection, analysis, interpretation of data, the writing of this article, or the decision to submit it for publication. The remaining authors declare that the research was conducted in the absence of any commercial or financial relationships that could be construed as a potential conflict of interest.

## Publisher’s Note

All claims expressed in this article are solely those of the authors and do not necessarily represent those of their affiliated organizations, or those of the publisher, the editors and the reviewers. Any product that may be evaluated in this article, or claim that may be made by its manufacturer, is not guaranteed or endorsed by the publisher.
