# Supplementary material for: Spatial Resolution of Suprachoroidal–Transretinal Stimulation Estimated by Recording Single-Unit Activity From the Cat Lateral Geniculate Nucleus
Source: Front Neurosci. 2021 Oct 13;15:717429. doi: 10.3389/fnins.2021.717429 (PMC8549691; doi:10.3389/fnins.2021.717429)
Supplement: Supplementary file 1 [file Data_Sheet_1.pdf]

Supplementary Material for

Miyoshi T., Morimoto T., Sawai H. and Fujikado T.

Spatial resolution of suprachoroidal-transretinal stimulation estimated by recording single-unit activity from the cat lateral geniculate nucleus.

Frontiers in Neuroscience

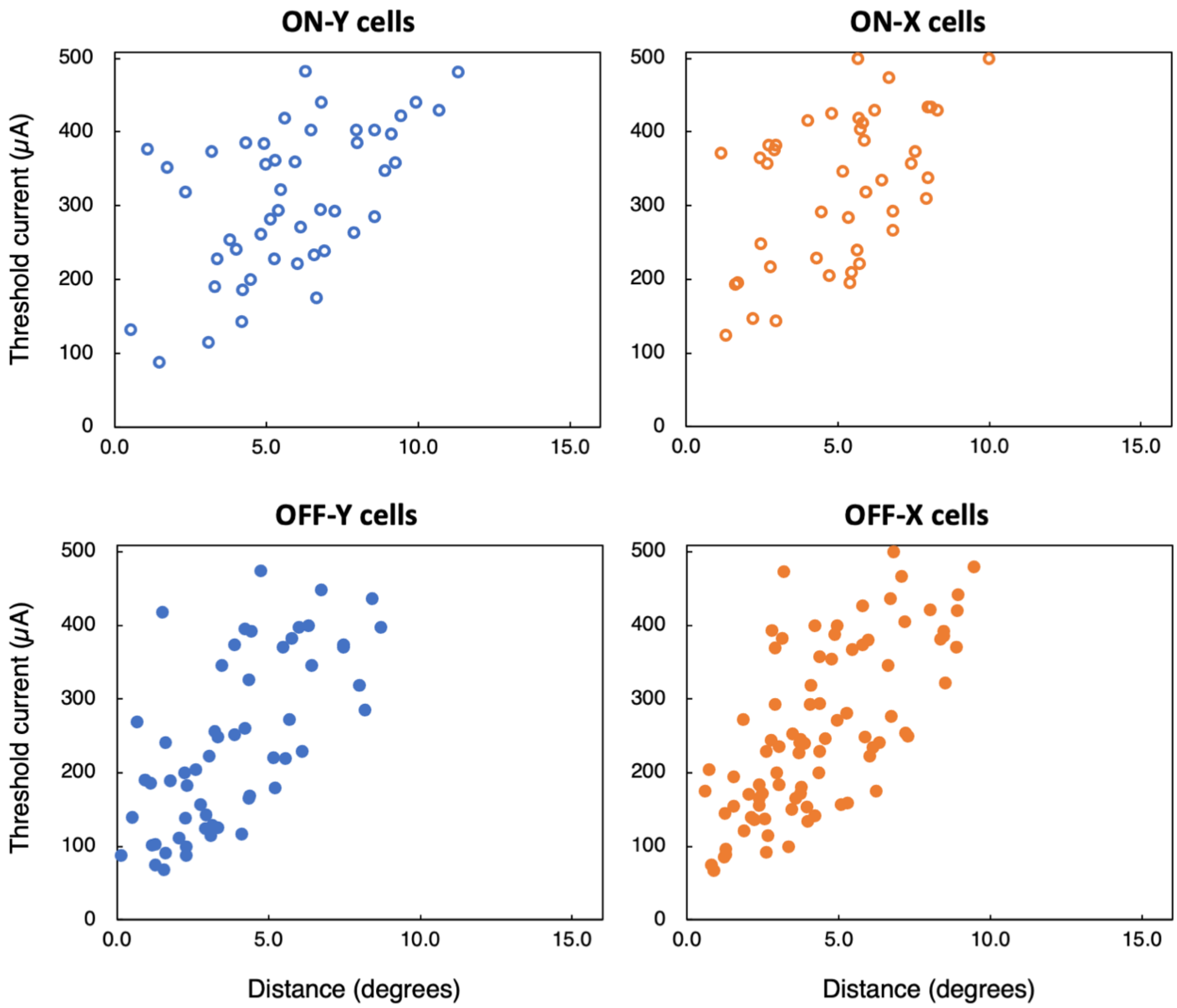

Supplementary Figure 1. The distribution of thresholds classified by the subtypes of neurons, On-center cell/Off-center cell and Y-cell/X-cell. Combined across panels the data is the same as in Figure 3.

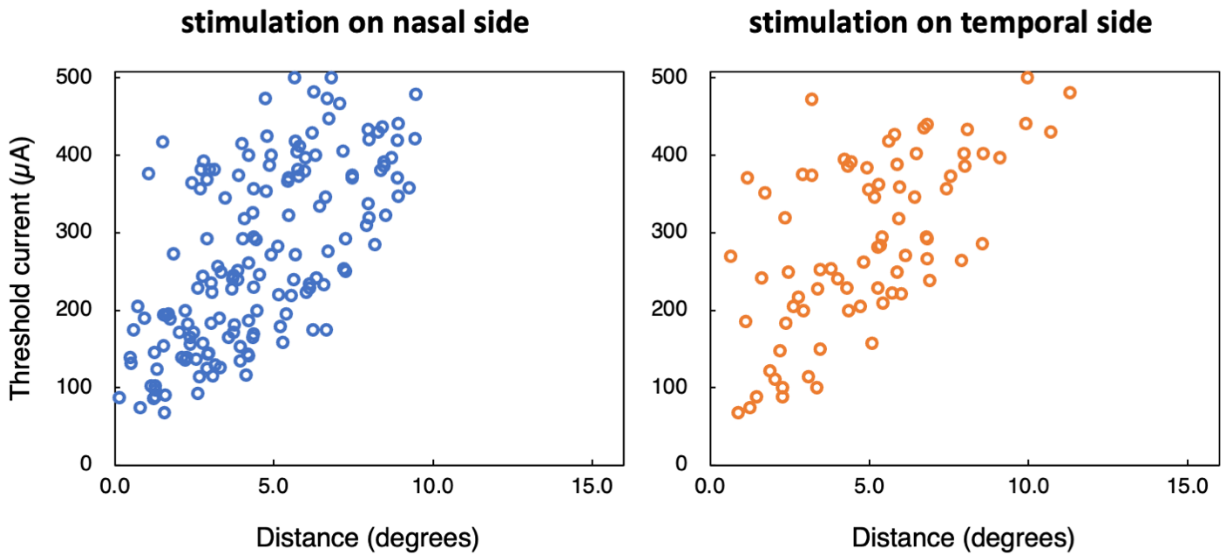

Supplementary Figure 2. The distribution of thresholds classified by the stimulation on the nasal side from the receptive field center and the stimulation on the temporal side. The stimulation on nasal side had the possibility to stimulate the passing axon, because the simulation electrode was implanted into temporal retinal. Combined across panels the data is the same as in Figure 3.

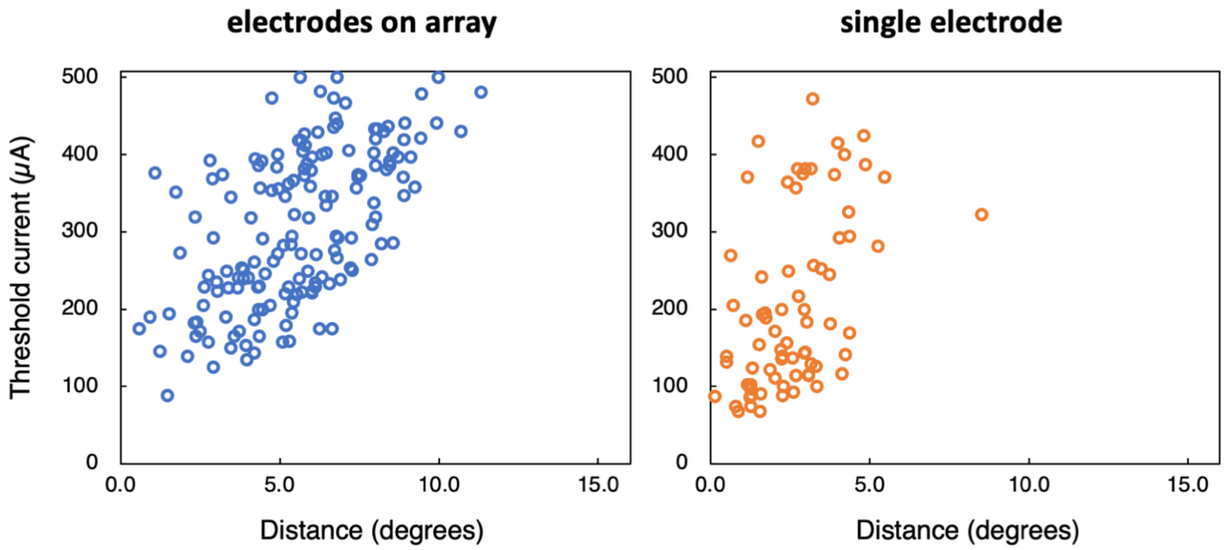

Supplementary Figure 3. The distribution of thresholds classified by the types of the stimulation electrode, electrode array with 9 electrodes or single electrode. Combined across panels the data is the same as in Figure 3.

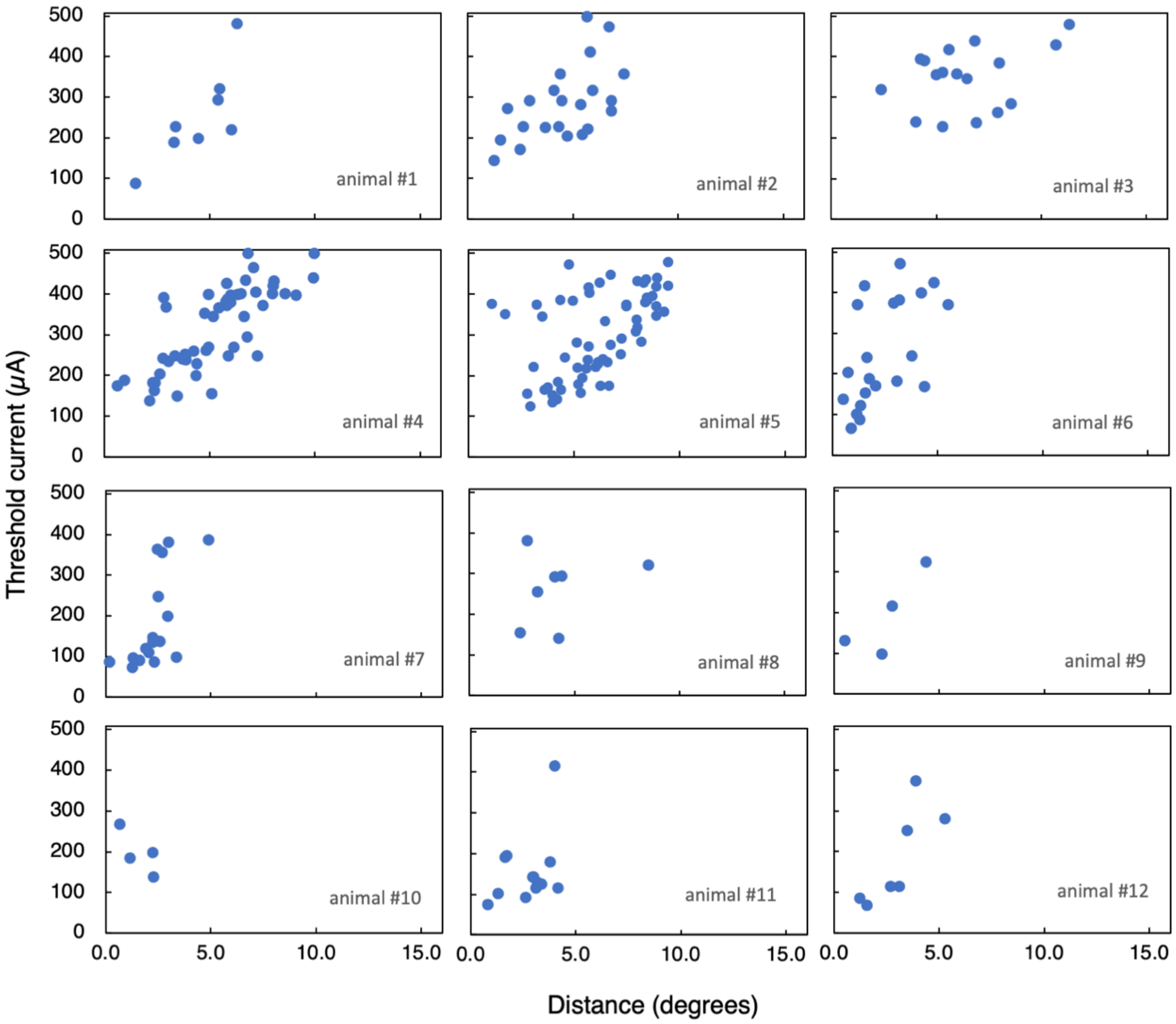

Supplementary Figure 4. The distribution of thresholds classified by the experimental animals. Combined across panels the data is the same as in Figure 3.

Factors	Standardized regression coefficient (β)	Significance probability (p-value)	Regression coefficient (γ)
Distance between RF and SE	9.377	<0.001	27.567
Cell type (Y-cell/X-cell)	1.450	0.148	16.812
Cell type (On-center cell/Off-center cell)	−2.396	0.017	−31.427
Relative position of SE to RF (nasal/temporal)	−0.349	0.727	−4.618
Type of SE (single/array)	−0.843	0.400	−12.591
Multiple correlation coefficient (R)		0.659	
Adjusted R^2		0.422	

Supplementary Table 1. Summary of multiple regression analysis for threshold (based on forced entry method). Among the five factors as explanatory variables, the effectiveness differed significantly between the stratified groups of two factors, namely the distance and the cell type (On-center cell/Off-center cell). There were no significant differences between the stratified groups in the other three factors, namely, the cell type (Y-cell/X-cell), the relative position of stimulation electrode (nasal/temporal to RF), and the electrode type (single/array electrode). The coefficient of determination was 0.659 and coefficient of determination with adjusted degrees of freedom was 0.422. RF, receptive field; SE, stimulation electrode.

Factors	Standardized regression coefficient (β)	Significance probability (p-value)	Regression coefficient (γ)
Distance between RF and SE	11.756	<0.001	28.952
Cell Type (On-center cell/Off-center cell)	−2.346	0.020	−28.340
Multiple correlation coefficient (R)		0.653	
Adjusted R^2		0.422	

Supplementary Table 2. Summary of multiple regression analysis for threshold (based on stepwise method). A significant prediction formula was performed, the coefficient of determination and coefficient of determination with adjusted degrees of freedom did not noticeably improve.
